# Errors in Table 1 and Table 3

**DOI:** 10.1001/jamahealthforum.2024.1974

**Published:** 2024-06-28

In the Original Investigation titled “Budget Impact Analysis of Circulating Tumor DNA Testing for Colon Cancer in Commercial Health and Medicare Advantage Plans”^1^ published on May 31, 2024, in *JAMA Health Forum*, there were errors in Table 1 and Table 3. In Table 1, there were some errors in the proportions by age. In Table 3, cells containing “1 [Reference]” have been corrected to “0 [Reference].” This article was corrected online.
